# Transregional Study Highlighting the Increasing Burden of Urology Cancer Multidisciplinary Team Meetings Around the UK

**DOI:** 10.7759/cureus.48501

**Published:** 2023-11-08

**Authors:** Zain Kasmani, Wail Mohamed, Zain Siddiqui, Saddek Boksh, Shaswath Ganapathi, Zakaria Saidani, Don S Wijayasuriya, Jack Donati-Bourne

**Affiliations:** 1 Urology, Birmingham City Hospitals, Birmingham, GBR; 2 Urology, Royal Shrewsbury Hospital, Shrewsbury, GBR; 3 Urology, Countess of Chester Hospital National Health Service (NHS) Foundation Trust, Manchester, GBR; 4 Urology, Sandwell and West Birmingham Hospitals, Birmingham, GBR; 5 Urology, Newport Hospital, Wales, GBR

**Keywords:** quality improvement and patient safety, standard of care, management, waiting list, uk - united kingdom, cancer, multidisciplinary care team

## Abstract

Introduction

The urology multidisciplinary team meeting (MDT) is the key weekly meeting that allows the opportunity to review results and discuss management plans for all urological cancers within a department. As populations age and cancer detection and management improve, the demand for the MDT will increase. We conducted a collaborative transregional study within the UK to evaluate the current workload on the urology MDT.

Methods

The study was divided into two parts: a multicenter retrospective audit and a snapshot survey. Three UK hospitals in Birmingham, Liverpool, and Cardiff were recruited into the multicenter study. Each hospital provided full MDT lists for all weekly meetings between August 2017 and 2022. Retrospective data gathered included the number of patients discussed per week, the average age of patients per week, the time allocated to their weekly MDT, and the total number of consultants in the department. The second part of the study involved the distribution of an online questionnaire to urologists across the UK to obtain a snapshot picture with the above parameters.

Results

Snapshot data from 34 different UK hospitals showed MDT length ranged from 1-6 hours, patients discussed ranged from 10-90 per week, and the maximum average discussion time was 3.8 minutes per case. Furthermore, 76% (N = 28/37) of respondents said unnecessary cases were discussed. Varied suggestions were provided on how the MDT could be improved.

Multicenter five-year data showed a rise in mean total numbers of patients discussed per week in all centers: a 34.8% increase in Birmingham (from 34.5 patients to 46.5 patients), a 23.5% increase in Liverpool (27.2 patients to 33.6 patients), and a 38.8% increase in Cardiff (22.7 patients to 31.5 patients). Hours per meeting were Birmingham (2), Liverpool (3), and Cardiff (4), which meant the average minutes per patient discussion were Birmingham (2.6), Liverpool (5.4), and Cardiff (7.6).

Conclusion

There is a rapidly rising trend across UK regions for the number of patients being discussed in the urology MDT meeting. The MDT structure and function across the country are highly variable. There is consensus that the MDT discusses cases that are unnecessary, and this has been recognized for many years. Widespread implementation of the latest MDT management guidelines is urgently required to ensure MDT meetings are able to function effectively and efficiently into the future.

## Introduction

The multidisciplinary team (MDT) meeting has been recognized as a cornerstone for cancer management within the United Kingdom (UK) since the publication of the National Cancer Plan in 2000 [1]. A urology MDT meeting brings together cross-specialty healthcare professionals in a collaborative manner, focusing their collective expertise on a single case at a time to produce optimal decision-making for patient care. The MDT provides a planned platform for clinicians to rubber-stamp their cases, discuss challenges, and seek second opinions. Furthermore, the MDT safeguards patient care by preventing a single individual from making complex, potentially life-altering decisions without approval and support.

The UK has an aging population [2]. This, combined with rapid improvements in the detection and management of cancer, results in a greater number of people being diagnosed and living with cancer into old age. Indeed, the National Health Service (NHS) not only recognizes this but aims to make it a reality. The ‘NHS Long Term Plan’ for cancer states that by 2028, 55,000 more people each year will survive their cancer for five years or more [3]. The care of these patients, current and future, will ultimately go through the MDT, which will see an increasing strain on its resources. Subjective reporting and our own observations suggest that the MDT is already stretched and struggling to cope with demand. There is, therefore, mounting concern regarding the quality of care that MDT will be able to deliver in the future.

Our aim was to study the current pressures placed on the urology MDT around the United Kingdom (UK). We aimed to highlight the current MDT workload across the UK and, furthermore, demonstrate whether there has been any significant increase in workload over the years.

## Materials and methods

Two separate studies were conducted: a snapshot questionnaire and a multicenter retrospective collaborative audit.

Snapshot questionnaire: A questionnaire was created using the online platform Google Forms [4]. This questionnaire was distributed to urology doctors working across the UK via email and personal contacts. A total of six open-ended questions and one yes/no question were asked (Table 1). To be eligible to respond, those surveyed must have worked in their current position for at least three months. The responses were anonymous. Data was captured over a seven-day period in October 2023. The maximum number of hours allocated to the MDT meeting divided by the total number of cases was used to calculate an average maximum possible 'minutes per case' discussion time per hospital.

**Table 1:** Summary of questions asked within the Google Forms questionnaire

Question	Open Ended (O)/Multiple Choice (M)
Which UK hospital do you/did you work in?	O
How many hours are allocated to the MDT each week?	O
How many patients, on average, are discussed per MDT meeting?	O
How many Urology Consultants, on average, attend the MDT?	O
In your opinion, are unnecessary cases discussed at your MDT?	M (Yes / No)
If you answered 'yes' to the above question, what percentage of cases (%) do you believe are unnecessarily discussed?	O
In your opinion, how could the Urology MDT be improved?	O

Multicenter audit: A multicenter transregional audit was conducted across three hospitals in the UK, based in the following cities: Birmingham, Liverpool, and Cardiff. In each hospital, a lead auditor was appointed and collected the data from their respective department’s weekly MDT meeting for the years August 2017 through August 2022. This was done by recalling the MDT meeting data, which each department stores electronically on their own systems. This generated approximately 250 MDT dates per hospital over the course of five years. Expected MDT meeting cancellations due to bank holidays were accounted for and labeled with a 'U' for 'unavailable'. Data extracted included the total number of patients discussed per week and their calculated average age. Each hospital also provided information on the duration of the MDT and the number of consultants present. The data for all three centers was assembled into a master spreadsheet and subsequently used to calculate the average yearly numbers of patients discussed, their average age, and the average discussion time per case. 

## Results

Snapshot survey

Thirty-eight responses were received in a seven-day period from urologists across the UK. One response was removed, given that the hospital name was not recognized. Three of the hospitals were mentioned twice, giving 34 different hospitals with widespread geographical distribution (Table 2).

**Table 2:** Summary of all hospitals from which data was gathered

The Royal Gwent Hospital
Royal Stoke
Sandwell General Hospital X 2
Shrewsbury and Telford Hospitals NHS Trust X 2
Peterborough city hospital
Sherwood Forest Hospitals NHS Trust
Freeman Hospital Newcastle Upon Tyne
Royal Wolverhampton NHS trust
University Hospital Coventry and Warwickshire
Bedford Hospital
Whittington Hospital
Manchester Foundation Trust
United Lincolnshire Hospitals NHS Trust
Cambridge University Hospitals
East Lancashire Hospitals Trust
Leighton Hospital
Cheltenham General Hospital X 2
Queen Elizabeth University Hospital, Glasgow
University College London
West Herts NHS Trust
Luton & Dunstable
Pinderfields Hospital
Countess of Chester
Darent Valley Hospital
Kingston hospital
Alexandra hospital
University Hospital Coventry & Warwickshire
Ninewells Hospital
Hereford County Hospital
Dudley Group NHS Foundation Trust
Liverpool University Hospitals
Queen Elizabeth Hospital, Kings Lynn
Addenbrookes
George Eliot hospital

**Figure 1:**
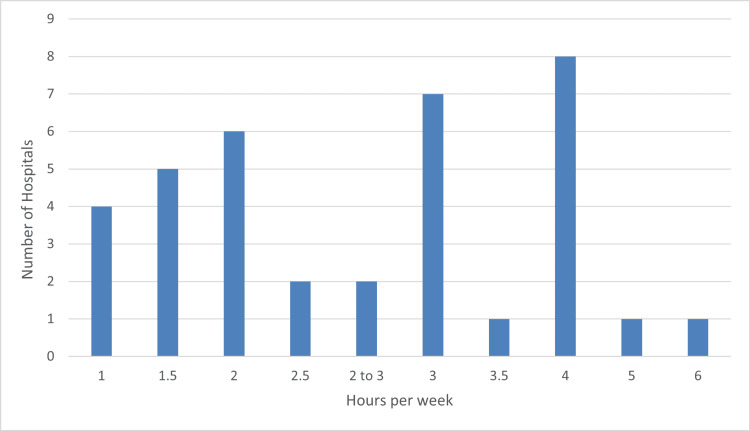
Bar chart showing the range of hours allocated to the multidisciplinary team meeting per week with an associated number of hospitals

There was a significant variety in the duration of the MDT meetings across the 37 valid responses. The meetings range from 1 hour to 6 hours per week; the mode was 4 hours, and the average was 2.7 hours per week. An anonymized bar chart representing this is shown below (Figure 1).

**Figure 2:**
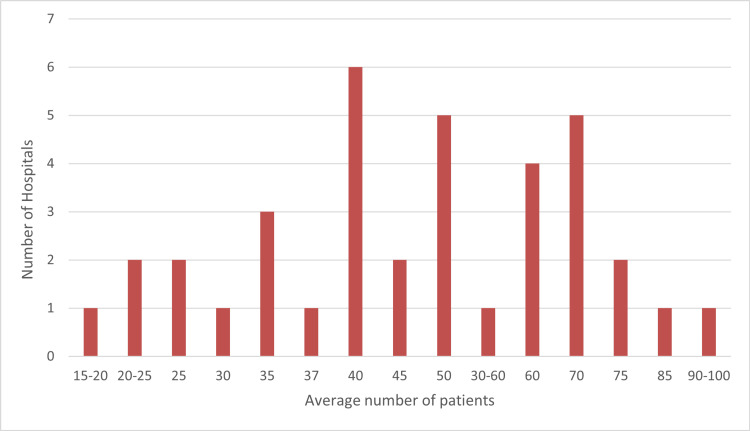
Bar chart showing the average number of patients discussed per week with an associated number of hospitals

Figure 2 shows the anonymized average number of patients discussed per week, as reported directly by responders through free-text answers. The range is from 15 to 90 patients per week, with a maximum of 40 patients.

**Figure 3:**
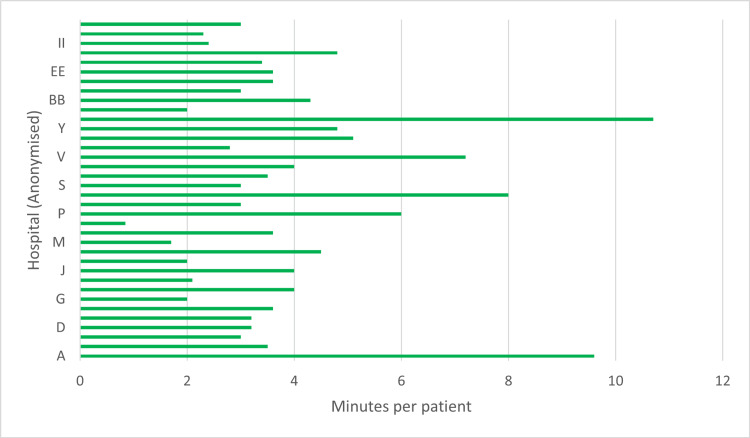
Bar chart summarizing the average maximum possible number of minutes allocated to each patient discussion per week in each hospital

The maximum time allocated to the MDT meeting was divided by the minimum number of average patients to give a maximum possible number of minutes allocated to each patient discussion, rounded to one decimal place. The range is 0.86 to 10.7 minutes per case, with an average maximum of 3.8 minutes across 37 responses. The range of anonymized responses is shown in Figure 3.

When asked if they believed unnecessary cases were discussed in MDT, 76% (N = 28/37) of respondents agreed there were. The percentage range of unnecessary cases discussed ranged from 5% to 70% of cases, with 10% being the most common answer (N = 12/37) and 20% being the median answer.

When asked how the urology MDT meeting could be improved, 33 responses were received with a variety of suggestions (Table 3). While various opinions were given, common themes included the words ‘streamlining’ and ‘preparation’ as well as the idea that straightforward cases do not need discussing. 

**Table 3:** Range of written responses to the question on how multidisciplinary team meetings could be improved.

Prior preparation, protocolizing cases, filter unnecessary cases
Streamlining the unnecessary cases by the MDT chair
Juniors not having to prep the notes in their own time
Registrars' involvement in case presentations and decision making will optimize the educational value of MDTs. Responsible consultants or clinicians who know the patients being discussed can guide the MDT to make bespoke recommendations.
By following standard protocol for usual cases and making a definitive plan complex cases. Cases should be presented by the person who has seen the patient rather than someone else.
MDT notes should automatically include previous MDT discussions
Triage, active participation of middle grades
By not discussing cases where the outcome is obvious
F2F rather than Teams, in house discussion instead of linking in with many sites
More time to look at some cases in greater detail as might be required
More specialties join... Nurses play a more active role
Limit postop histology discussion
Prepare the notes in advance and have a specific question that needs answering.
Subdivide into subspecialties – i.e., kidney, prostate, bladder. Combine the MDT for difficult cases. Would allow time for admin after MDT to be completed
Streamlining and register straightforward cases
Have a triage for MDT patients and all having a dedicated MDT session including juniors to learn from MDT
A lot of preparation can take place to minimize the time spent per patient
Given adequate time to discuss cases
MDT proforma filled out by referring team to reduce time searching for clinics information during MDT.
MDT streamlining
Cases should be presented by REG/CNS. Consultants then discuss and give their views. REG/CNS documents all discussion and outcome.
Better streamlining of referrals
Proper job planned session for urology and non-Urology colleagues, good admi support along with cancer care coordinator. Also, guidance and protocol documents for common and straightforward pathologies should be available for quick actions in MDT, adding appropriate clinical information on MDT form and with feedback to requester
Educating referrers on straight forward cases to avoid referrals as decision can be made directly
More time needed; I feel due to lack of time we rich to the conclusions. Better prepping makes a difference
Better clinical summary on clinical letters, ideally similar to oncology or medical letters
Time for Pre MDT prep
Time consuming way by vetting every request
The use and adherence of a standardized referral proforma within regional cancer network for sMDT
Having a team engaged in the cases. Develop a pathway for prostate biopsies so only key biopsies are discussed.
Paperless MDT, use of AI, improving radiology reporting quality

The data on the number of consultants present at the meeting has been discarded. It was made clear from the responses that some respondents recorded the number of urology consultants present, while others recorded the total number of consultants present, regardless of specialty. This information cannot, therefore, be reliably compared and analyzed. 

Multicenter five-year data

Each site provided approximately 250 MDT dates from August 2017 to August 2018. This consisted of one meeting a week for five years, not accounting for national holidays. For each meeting date, the number of patients discussed was noted, and the average age of patients calculated. This information was then used to provide an average for each year across all three hospitals involved, summarized in Table 3. There was an increase in the number of patients discussed year-on-year across each site. The average increase from 2017 to 2022 was 34.8% (from 34.5 patients to 46.5 patients) in the Birmingham hospital, 23.5% (27.2 patients to 33.6 patients) in the hospital in Liverpool, and 38.8% (22.7 patients to 31.5 patients) in Cardiff.

The number of hours allocated to the MDT meeting was 2 hours in Birmingham, 3 hours in Liverpool, and 4 hours in Cardiff. For the year 2022, this gives a maximum average discussion time per patient of 2.6 minutes, 5.4 minutes, and 7.6 minutes, respectively.

The average age (Table 4) of the patients discussed was also noted. There was no significant increase in the age of the patients discussed over the past five years. The average age of patients discussed in 2022 across all three hospitals was 68.6 years.

**Table 4:** Summary table showing the increase in the average number of patients discussed each year per hospital as well as their average age

	Birmingham		Liverpool		Cardiff	
Year	Patients	Age	Patients	Age	Patients	Age
2017	34.5	68.2	27.2	66.4	22.7	69.9
2018	35.2	67.1	28.1	67.0	23.0	70.3
2019	34.4	66.8	29.3	67.3	22.9	69.0
2020	34.2	66.8	28.7	67.1	19.9	68.9
2021	41.7	67.3	32.7	67.5	28.3	69.0
2022	46.5	67.1	33.6	68.4	31.5	70.3

## Discussion

The snapshot aspect of this study demonstrates the range in MDT meeting time and patient numbers across the UK in 2023, but it does carry limitations. The study was sent to urology doctors (specialist registrars and consultants) working within the UK, as this group of healthcare professionals most frequently attend the MDT meeting and are best placed to provide accurate answers. However, the information provided per hospital is from a single source at a given point in time, and no objective raw data was required in the answers, meaning there is a possibility of recruitment and recall bias. In order to mitigate this, responders were asked to answer only if they had worked in their hospital for at least three months. This would allow enough time to gather a representative sample. However, there was no method to validate their answers or prevent responders from answering if they had worked less than three months. The data was gathered from 34 different hospitals in the UK. There are currently 215 trusts within the UK [5]. Over 140 of them provide urology services [6], and each trust can be made up of multiple hospitals, meaning the sample size only makes up a small proportion of UK urology hospitals. While this sample size is small, the results are comparable to the studies discussed below and fit with national trends. The multicenter data is made up of approximately 750 MDT dates across three hospitals over five years-two hospitals within England and one in Wales. Although the retrospective data covers a long period and is objective, the small sample size may not represent trends in Scotland and Northern Ireland. 

This study has highlighted the variation in structure and function of the Urology MDT that exists in 2023, as showcased by the range in MDT meeting time, the number of cases discussed, and consequently the overall maximum discussion time per patient (3.8 minutes per case average). These findings are not new. Raine et al. carried out a prospective observational study in 2014, observing 370 MDT meetings, and concluded that ‘substantial diversity exists in the purpose, structure, processes, and content of MDT meetings’ [7]. Similarly, Cancer Research UK carried out a smaller study of 624 MDT discussions across 24 meetings in 10 sites and found the average length of discussion to be 3.2 minutes per case, and over half of cases were discussed in less than 2 minutes [8]. NHS England recognizes the fact that the MDT meeting as it was originally designed is no longer suited to cope with the demand for cancer care placed on the National Health Service (NHS) [9]. The key recommendation states that streamlining can be facilitated by following ‘Standards of Care’ (SoC). An SoC is a recognized, gold-standard management pathway through which a patient can be placed at a recognized point in their disease. Straightforward cases can follow these pathways and need not be discussed in a resource-intensive meeting, which should be preserved for the more complex cases. However, our snapshot survey shows that 76% (N = 28/37) of respondents currently believe unnecessary cases are being discussed, and the median number of unnecessary cases was 20% (median) of the meeting total. Many respondents stated that 'streamlining' was needed. Similarly, a study by review by Warner et al. based on data from 2017 showed that 87% of urology MDT members felt some patients could be managed outside of the MDT, and this view was also held by the lung (78%), breast (75%), and colorectal (64%). The five-year gap between that data and the results of this study suggests that the above guidance is either not known about or not being followed appropriately. 

Our multicenter transregional study highlighted two key points. First, there is an average 32.5% increase in the number of cases being discussed across the three hospitals in different parts of the UK over a five-year period. Although the overall trend is up, the year-on-year data suggests the greatest jump occurred between 2020 and 2021. This is possibly a reflection of normal services resuming following the national lockdown secondary to the COVID-19 pandemic. Normal workload combined with the backlog of undiagnosed and undertreated cases could be responsible for this increase. UK national statistics support the data in this study, emphasizing that the COVID-19 pandemic has exacerbated a pre-existing burden, not caused it. Hospital waiting lists for treatment have been increasing since 2014, and the percentage of patients failing to meet cancer treatment deadline targets has been increasing steadily over the same period [11]. Around 357,000 people in the UK were diagnosed with cancer in 2014, and this figure is expected to increase by 2035 to 500,000 per year [9]. The above data and predictions show the workload of the MDT has increased and will likely continue to do so in subsequent years.

The average age of patients discussed in 2022 across the three hospitals was 68 years old. By 2040, national projections suggest the number of over-85s will double and that 1 in 5 adults in England will be living with a major illness [12]. Other studies predict an upward trend in those living with complex multi-morbidity, defined as more than four diseases [13]. Increasing age, associated morbidity, and major illnesses add complexity to patient management, requiring more nuanced discussion and tailored treatment plans. These types of discussions mandate effort and time. Increasing the allocated time for MDT meetings is a shortsighted solution, and certainly prolonged meetings are to be avoided in order to prevent decision-making fatigue [14]. This showcases the need to urgently restructure the MDT to cope with this burden and divert simpler cases to approved management pathways where appropriate.

The results of this study, combined with other research and national data, demonstrate the MDT is under significant strain. This problem is already recognized, and guidance is available to facilitate improvement. This study suggests that significant work remains to safeguard the future success of the MDT and, consequently, patient care. For example, Lamb et al. recognized the need for training in MDT management and, as such, developed courses to enable this to take place [15]. Education, use of SoCs, organ-specific meetings, uptake of novel technology, and continuous quality improvement are required to streamline the MDT meeting and ensure the quality is consistent across the UK. Urology, along with all specialties dealing with cancer, should rapidly embrace new ways of managing their MDT in order to effectively cope with present and future demands.

## Conclusions

Our combined data has shown that the urology MDT meeting varies greatly in structure and function across the country. There is further evidence that the workload on the MDT has been increasing steadily over the last five years and will continue to do so. These concerns are recognized by other published works. Solutions have been proposed to streamline the MDT, but according to this study, results have yet to manifest across the UK as a whole. Rapid adoption of new guidelines, education, training, and restructuring are required to ensure the MDT meeting remains an effective and safe resource in the management of cancer in the UK. 

## References

[1] (2023). Department of Health: The NHS plan: a plan for investment, a plan for reform. https://webarchive.nationalarchives.gov.uk/ukgwa/+/www.dh.gov.uk/en/publicationsandstatistics/publications/publicationspolicyandguidance/dh_4002960.

[2] (2023). Office for National Statistics: Living longer: how our population is changing and why it matters. https://www.ons.gov.uk/peoplepopulationandcommunity/birthsdeathsandmarriages/ageing/articles/livinglongerhowourpopulationischangingandwhyitmatters/2018-08-13.

[3] (2023). NHS: Overview and summary. https://www.longtermplan.nhs.uk/online-version/overview-and-summary/.

[4] Raine R, Wallace I, Nic a’ Bháird C (2014). Improving the effectiveness of multidisciplinary team meetings for patients with chronic diseases: a prospective observational study. NIHR Jr Lib.

[5] (2023). The King's Fund: Key facts and figures about the NHS. http://www.kingsfund.org.uk.

[6] (2023). Interweave Healthcare: How many hospitals in the UK. https://gettingitrightfirsttime.co.uk/wp-content/uploads/2018/07/GIRFT-Urology.pdf.

[7] MEETING PATIENTS’ NEEDS (2023). Cancer Research UK: Meeting patient's needs. https://www.cancerresearchuk.org/sites/default/files/executive_summary_meeting_patients_needs_improving_the_effectiveness_of_multidisciplinary_team_meetings.pdf.

[8] (2023). NHS: Streamlining multi-disciplinary team meetings. https://www.england.nhs.uk/wp-content/uploads/2020/01/multi-disciplinary-team-streamlining-guidance.pdf.

[9] (2023). UK Parliament: NHS key statistics. July.

[10] Warner R, Hoinville L, Pottle E, Taylor C, Green J (2021). Refocusing cancer multidisciplinary team meetings in the United Kingdom: comparing urology with other specialties. Ann R Coll Surg Engl.

[11] (2023). The Health Foundation: Health in 2040: interactive chart projections. https://www.health.org.uk/news-and-comment/charts-and-infographics/health_in_2040.

[12] (2023). East of England Cancer Alliances: MDTM transformation. https://www.canceralliance.co.uk/professionals/early-diagnosis/mdtm-transformation.

[13] Kingston A, Robinson L, Booth H, Knapp M, Jagger C (2018). Projections of multi-morbidity in the older population in England to 2035: estimates from the Population Ageing and Care Simulation (PACSim) model. Age Agi.

[14] Soukup T, Gandamihardja TA, McInerney S, Green JS, Sevdalis N (2019). Do multidisciplinary cancer care teams suffer decision-making fatigue: an observational, longitudinal team improvement study. BMJ Open.

[15] Lamb Lamb, B.W. B.W., Linton Linton, K.D. and Narahari, K K Baus oncology guidance for Implementing Streamlining in cancer MDT meetings: Selecting standards of care and operational considerations. Jr Clin Uro.

